# Salvaging a Failed PreserFlo MicroShunt Using a Telescopic Tube‐in‐Tube Connection Technique With an EyePlate Implant: A Case Report

**DOI:** 10.1002/ccr3.72072

**Published:** 2026-02-16

**Authors:** Qian Wei, Frank G. Holz, Karl Mercieca

**Affiliations:** ^1^ Department of Ophthalmology University of Bonn Bonn North Rhine–Westphalia Germany

**Keywords:** EyePlate‐200, glaucoma drainage device, PreserFlo MicroShunt, revision surgery, tube‐in‐tube connection

## Abstract

The PreserFlo MicroShunt is a subconjunctival glaucoma drainage device designed to reduce intraocular pressure (IOP) via a minimally invasive approach. However, device failure due to bleb fibrosis remains a significant clinical challenge, especially when initial revision surgery is unsuccessful. We present a 75‐year‐old male with pseudoexfoliation glaucoma who previously underwent PreserFlo MicroShunt implantation and a subsequent revision procedure at an outside institution. Due to persistently elevated IOP and progressive visual decline, he was referred to our center for further management. A novel telescopic tube‐to‐tube connection was performed between the existing PreserFlo MicroShunt and a new EyePlate‐200 implant, utilizing and preserving the original scleral tunnel. Postoperative IOP decreased from 40 to 10 mmHg and remained stable without complications during a 6‐month follow‐up. A surgical video is provided as Video 1. This case illustrates a novel telescopic tube‐to‐tube connection technique to restore aqueous outflow in cases of PreserFlo MicroShunt failure. By preserving the original scleral tunnel, this approach may offer a minimally invasive alternative to complete device explantation or conventional tube shunt surgery.

## Key Clinical Message

A telescopic tube‐in‐tube connection between a failed PreserFlo MicroShunt and an EyePlate‐200 implant may offer a minimally invasive rescue technique that preserves the original scleral tunnel and avoids full device explantation.

## 1 Introduction

Glaucoma is a leading cause of irreversible vision loss globally, with elevated intraocular pressure (IOP) recognized as its most important modifiable risk factor [1]. Surgical intervention is often necessary when medical or laser therapy fails to maintain adequate IOP control. The PreserFlo MicroShunt (PMS, Santen Pharmaceutical Co, Osaka, Japan) is part of the growing category of less invasive bleb‐forming procedures [2]. It is a subconjunctival drainage implant designed for ab externo implantation, providing IOP reduction by directing aqueous outflow to a subconjunctival bleb [3]. It therefore differs from traditional “long tube” implants, also known as Glaucoma Drainage Implants (GDIs), by having much smaller inner and outer tube diameters and no bulky posterior plate [4].

Despite its less invasive nature and generally favorable outcomes, the PMS can fail due to fibrotic encapsulation of the distal bleb, resulting in increased outflow resistance and inadequate pressure control [5]. Tenon's capsule hyperplasia and local inflammatory responses to the subconjunctival implant material have also been implicated in bleb failure [6]. In such cases, revision with needling or open bleb revision is typically attempted, though outcomes are variable [7]. When revision is unsuccessful, clinicians are often left to choose between complete device removal and implantation of a conventional GDI such as the Ahmed or Baerveldt implant—both of which require new scleral tunneling and increased conjunctival dissection [8, 9].

Here, we report a novel rescue technique in a patient with failed PMS implantation and unsuccessful revision. Instead of removing the original device, we connected the PMS tube to a new EyePlate‐200 (Rheon Medical SA, Lausanne, Switzerland) using a telescopic tube‐in‐tube technique through the existing scleral tunnel. The outer diameter of the PMS (350 μm) slightly exceeds the internal lumen of the EyePlate (300 μm), necessitating beveling and dilation of the EyePlate tube to achieve a functional fit. This approach preserved ocular anatomy and minimized surgical trauma while effectively restoring outflow into the post‐equatorial subconjunctival space.

## 2 Case History and Examination

A 75‐year‐old male with a history of pseudoexfoliation glaucoma in the left eye had previously undergone PMS implantation approximately 2 months prior, followed by one revision surgery a few weeks later at an outside institution. Despite these interventions, IOP remained elevated (up to 40 mmHg), and uncorrected visual acuity (UCVA) in the left eye was approximately 20/100 Snellen, necessitating referral to our hospital for further surgical management. Given that a previous revision had already failed, further surgical management was considered.

## 3 Differential Diagnosis, Investigations and Treatment

**FIGURE 1:**
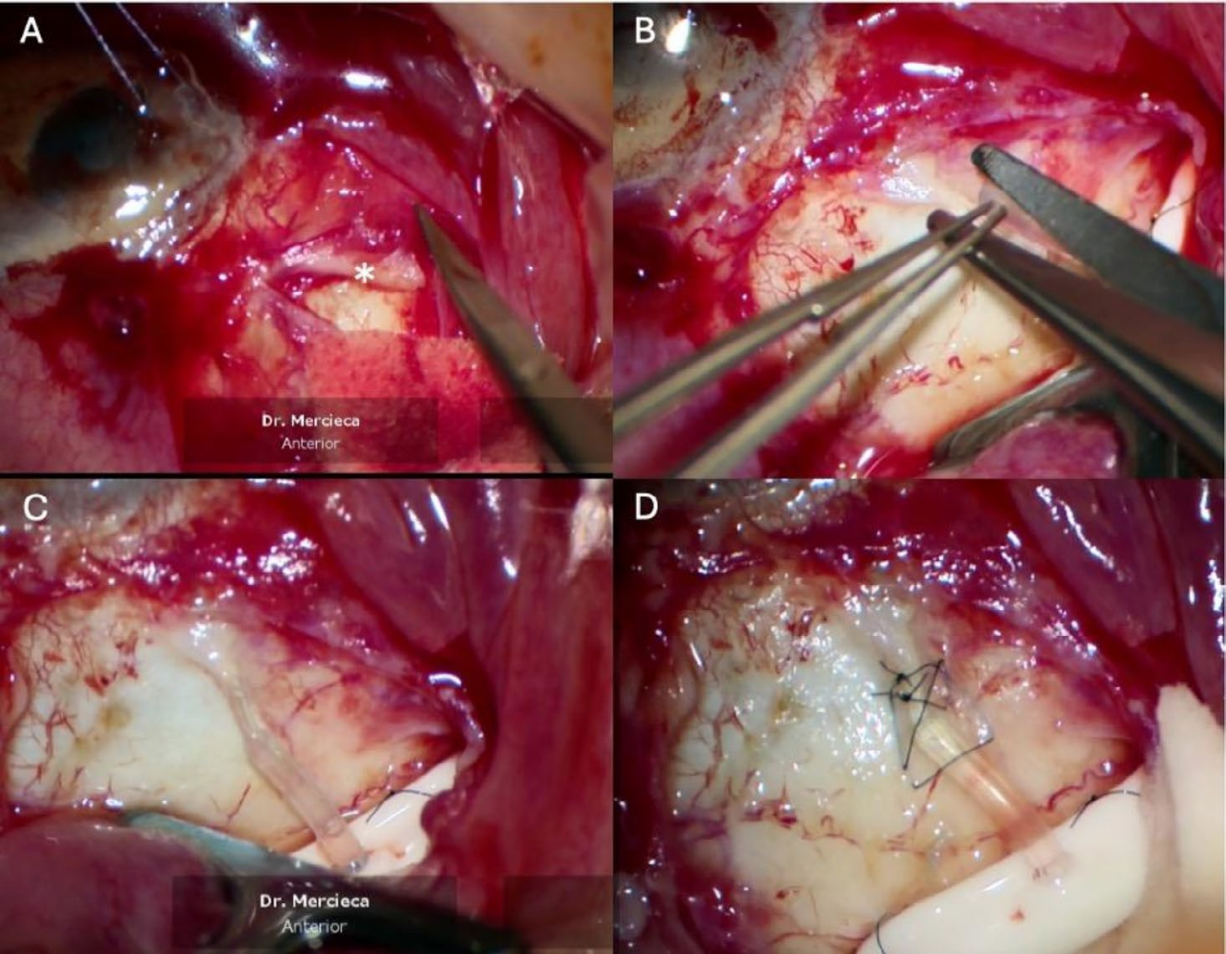
Key steps of telescopic connection between PMS and EyePlate. (A) Dense subconjunctival scarring (indicated by white asterisk) was dissected from around the distal portion of the original PMS. (B) The EyePlate tube was bevel‐cut to enlarge its lumen and facilitate telescopic insertion of the trimmed PMS tube. (C) The two tubes were telescopically connected under direct visualization. (D) The connection was anchored to sclera with 9–0 nylon sutures.

Given the persistently elevated IOP following both primary PMS implantation and a subsequent revision procedure, failure of distal aqueous outflow due to subconjunctival fibrosis was considered the most likely mechanism. As the prior revision attempt had already failed, a telescopic tube‐to‐tube connection was planned preoperatively as the next management step, with a view to tapping into the untouched, post‐equatorial, subconjunctival space. After a detailed discussion, informed consent was obtained for the proposed surgical procedure described below:

After sterile preparation, a 7/0 clear corneal Vicryl traction suture was applied followed by a limbal peritomy and careful posterior dissection of conjunctiva and Tenon's capsule in the area surrounding the PMS device. Dense subconjunctival scarring was observed around the distal portion of the PMS tube, and no aqueous flow could initially be detected (Figure 1A). However, further careful dissection of scar tissue around the shunt tip and flushing of the PMS with a 23G cannula re‐established good flow through the device. Instead of removing and replacing the PMS or creating a new scleral tunnel for GDI implantation, we elected to connect a new EyePlate‐200 implant to the existing PMS using a telescopic insertion technique. The EyePlate was positioned and secured approximately 12 mm posterior to the limbus to ensure post‐equatorial drainage. Because the external diameter of the PMS tube (350 μm) exceeds the internal lumen of the EyePlate‐200 (300 μm), the EyePlate tube was first shortened to the required length and then bevel‐cut at its distal end (Figure 1B) and gently dilated using a phaco spatula—with small controlled twisting movements to widen the lumen—allowing insertion of the trimmed PMS tube. The PMS tube was advanced into the EyePlate lumen using a controlled twisting motion, and a minimum telescopic overlap of approximately 2 mm was ensured to achieve a stable press‐fit connection, providing both a proper fit and a secure, leak‐free junction between the two tubes (Figure 1C). The junction was anchored to the sclera with 9–0 nylon suture (Figure 1D) and covered with a single‐layer fascia lata patch graft. Tenon's capsule and conjunctiva were then reapposed to their anatomical locations using 10/0 nylon suture. Aqueous outflow was checked and confirmed before conjunctival closure, and the system was flushed intraoperatively to ensure patency.

## 4 Conclusion and Results

Postoperative recovery was unremarkable. On postoperative day 1, UCVA remained approximately 20/100, and IOP decreased to 3–4 mmHg. By postoperative day 3, UCVA improved to approximately 20/50, and IOP stabilized at 1–2 mmHg without signs of hypotony or choroidal detachment. Fundus examination revealed peripheral retinal changes suggestive of early tractional detachment, which remained stable under observation. At the 6‐month follow‐up, IOP remained stable at 10 mmHg without the use of glaucoma medications, and UCVA was maintained at 20/50. The intraocular lens remained well centered, and no bleb‐related or tube‐related complications were observed during follow‐up. A detailed step‐by‐step surgical video is provided as Video 1.

**VIDEO 1:** Step‐by‐step surgical video demonstrating the telescopic connection of PMS to EyePlate using a tube‐in‐tube technique. Video content can be viewed at https://onlinelibrary.wiley.com/doi/10.1002/ccr3.72072.

## 5 Discussion

This case demonstrates that a telescopic tube‐to‐tube connection between a failed PreserFlo MicroShunt and an EyePlate‐200 implant can effectively restore aqueous outflow while preserving the original scleral tunnel and minimizing additional conjunctival dissection. PreserFlo MicroShunt failure typically reflects resistance to outflow at the distal end within the bleb, and revision attempts may be unsuccessful in eyes with significant scarring. When revision fails, more invasive alternatives are usually required. In our case, a minimally disruptive, anatomy‐preserving approach was preferred, consistent with principles highlighted in recent surgical innovation guidelines [10].

In this context, a stable telescopic junction was created by modifying the EyePlate tube to accommodate the existing PMS, permitting more posterior (post‐equatorial) redirection of aqueous flow, while preserving the original anterior chamber (AC) entry site. This approach aligns with minimally disruptive revision strategies, particularly in eyes with limited conjunctival reserve [11]. It also combines the advantages of a small diameter tube in the AC with the power of post‐equatorial, plate‐supported, subconjunctival drainage, which is typically achieved with the larger GDIs. Anchoring the junction with scleral sutures and covering it with a patch graft ensured both mechanical stability and watertight closure.

We are aware that other surgeons have explored connecting a PMS to a Baerveldt tube implant (Johnson & Johnson Vision, California, USA) in a similar telescopic manner, although to our knowledge, this technique has not been formally published and was only reported in the context of academic meetings. Compared with larger‐plate devices like the Baerveldt, the EyePlate‐200 features a smaller and more flexible implant footprint, enabling placement between—rather than underneath—the extraocular muscles. This minimizes tension on adjacent tissues and facilitates positioning, especially in anatomically crowded or previously operated eyes.

In addition, Brücher et al. recently described a modified approach for PAUL implantation in eyes with prior failed PMS, in which the same quadrant and original scleral tunnel were reused after explantation of the PMS [12]. Their method, while effective, still requires device removal and AC reinsertion and means that a larger external diameter tube is placed in the AC with potentially more risk of endothelial cell loss in the long term [13]. In contrast, our technique preserves the existing PMS in situ, avoids AC manipulation, thereby reducing the risk of endothelial trauma, hyphema, and postoperative inflammation associated with tube reinsertion—and leverages a direct tube‐to‐tube connection to restore outflow with minimal tissue disruption [14].

A separate report by Fritsche et al. demonstrated the insertion of a PMS segment into the lumen of a Baerveldt tube to limit flow in cases of hypotony [15]. However, that method served as a flow restrictor, whereas our approach aims to restore drainage after PMS failure, representing a fundamentally different surgical objective and mechanism.

To our knowledge, this is the first published case describing a direct device‐to‐device telescopic rescue of a failed PMS. This approach may be particularly useful in eyes with limited surgical space, conjunctival scarring, or failed PMS revision and highlights the potential for modular surgical strategies in glaucoma care.

While the short‐term results in this case were favorable, further studies are needed to assess long‐term efficacy, reproducibility, and safety. Nonetheless, this technique offers a promising addition to the surgical armamentarium for managing complex PMS failures.

## Author Contributions

**Qian Wei:** writing – original draft, writing – review and editing. **Karl Mercieca:** methodology, resources, supervision, writing – review and editing. **Frank G. Holz:** writing – review and editing.

## Funding

Qian Wei is supported by a doctoral scholarship from the Chinese Scholarship Council (Project ID: 202308510065). No specific funding was received for the design, execution, or publication of this work.

## Ethics Statement

The authors have nothing to report.

## Consent

Written informed consent was obtained from the patient for publication of this case report and accompanying images and video.

## Conflicts of Interest

The authors declare no conflicts of interest.

## Data Availability

All data generated or analyzed during this study are included in this published article.
